# Massive cardiac vegetation and cardiomyopathy in a 23‐year‐old rancher with brucellosis

**DOI:** 10.1002/ccr3.8177

**Published:** 2023-11-06

**Authors:** Seyed Mohsen Mirhosseini, Abdolhamid Bagheri, Mehran Lak, Zahra Ansari Aval, Mahdi Rezaei

**Affiliations:** ^1^ Cardiovascular Research Center Shahid Beheshti University of Medical Sciences Tehran Iran; ^2^ Department of Cardiology Modarres Hospital Shahid Beheshti University of Medical Sciences Tehran Iran; ^3^ Critical Care Quality Improvement Research Center Shahid Beheshti University of Medical Sciences Tehran Iran; ^4^ Faculty of Medicine Shahed University Tehran Iran

**Keywords:** brucellosis, endocarditis

## Abstract

**Key clinical massage:**

A 23‐year‐old rancher was admitted with the diagnosis of brucellosis. In evaluations, a massive vegetation in the aortic valve was seen. A combination of antibiotic therapy and cardiac surgery were performed, it seems this approach reduces mortality and complications.

**Abstract:**

Brucellosis (also known as “undulant fever,” “Mediterranean fever,” or “Malta fever”) is a zoonotic infection transmitted to humans from infected animals (cattle, sheep, goats, camels, pigs, or other animals) by ingestion of food products (such as unpasteurized dairy products) or by contact with tissue or fluids. It is the most frequent zoonosis globally and a major public health issue in many resource‐poor nations. Endocarditis is one of the rarest and most dangerous consequences of brucellosis. Additionally, the combination of endocarditis with cardiomyopathy increases its rarity. This condition is usually treated with a high level of suspicion Serological, clinical, and epidemiological data can all be used to make a diagnosis. The use of echocardiography aids in the early diagnosis. Due to the high risk of recurrence and the extent of tissue destruction brought on by Brucella, the majority of experts advise an early surgical intervention; nevertheless, other writers assert that low‐risk patients also require cautious therapy. In this article, we discuss the situation of a patient who underwent surgery and had Brucella endocarditis and heart failure. In conclusion, a combination of antibiotic therapy and cardiac surgery, reduces mortality and complications associated with Brucella endocarditis and improves patient quality of life.

## 1 INTRODUCTION

Brucellosis is a zoonotic and endemic systemic infectious illness that infects more than 500,000 individuals worldwide each year.^1^ Human infections are often caused by B. melitensis.^2^ In Iran and other Middle Eastern nations, brucellosis is an endemic disease, and its average incidence has been rising.^3^ Human brucellosis is a multisystem illness that is spread mostly by the use of unpasteurized milk or milk products.^4^ Fever, malaise, anorexia, headaches, muscular pains, arthralgia, exhaustion, and sweats can all be symptoms of systemic brucellosis. Some symptoms, such as recurring fevers, weariness, depression, arthritis, orchitis, endocarditis, and hepato‐splenomegaly, may last for a very long period. Also, Neurological involvement may be the only presenting characteristic.^4^, ^5^ Only 3% of human brucellosis morbidity is caused by cardiovascular problems, but they cause 80% or more of mortality from the disease. The majority of these cases are caused by endocarditis. Infected aneurysms and aortic ulcers are uncommon, but they can be fatal as well.^6^ According to some research, Brucella‐related endocarditis accounts for fewer than 1% of all brucellosis cases.^7^ In Brucella endocarditis, the aortic valve is most frequently impacted, whereas the mitral valve is infrequently affected.^8^ The best course of action for treating Brucella endocarditis is still up for debate, although the most generally used method is a mix of medicinal and surgical therapy.^9^ While it has been mostly suggested to remove the infected valves and replace them in addition to aggressive antibiotic treatment, it has been reported that only antibiotic treatment might also be adequate.^10^ In this report, we present a case of Brucella endocarditis. The patient was examined by transthoracic echocardiography (TTE) with the definitive clinical diagnosis made by Modified Duke criteria for the diagnosis of infective endocarditis. For the patients, surgery was planned following initial medical therapy. The patient had a good outcome after the operation.

## 2 CASE PRESENTATION

A 23‐year‐old rancher approached a hospital with an insidious fever and generalized pain complaint. He also mentioned weakness and lethargy, anorexia, and headache. He did not have any history of previous diseases or family history. In the examination he had Palpitations, the spleen, and cervical lymph nodes were palpated with increased size (spleen was palpated on left costal margin). Also, he had Shortness of breath, especially during exertion. He did not have chest pain, edema, orthopnea, and cough. In heart auscultation, S3 gallop rhythm was heard.

### 2.1 Diagnostic assessment

**FIGURE 1:**
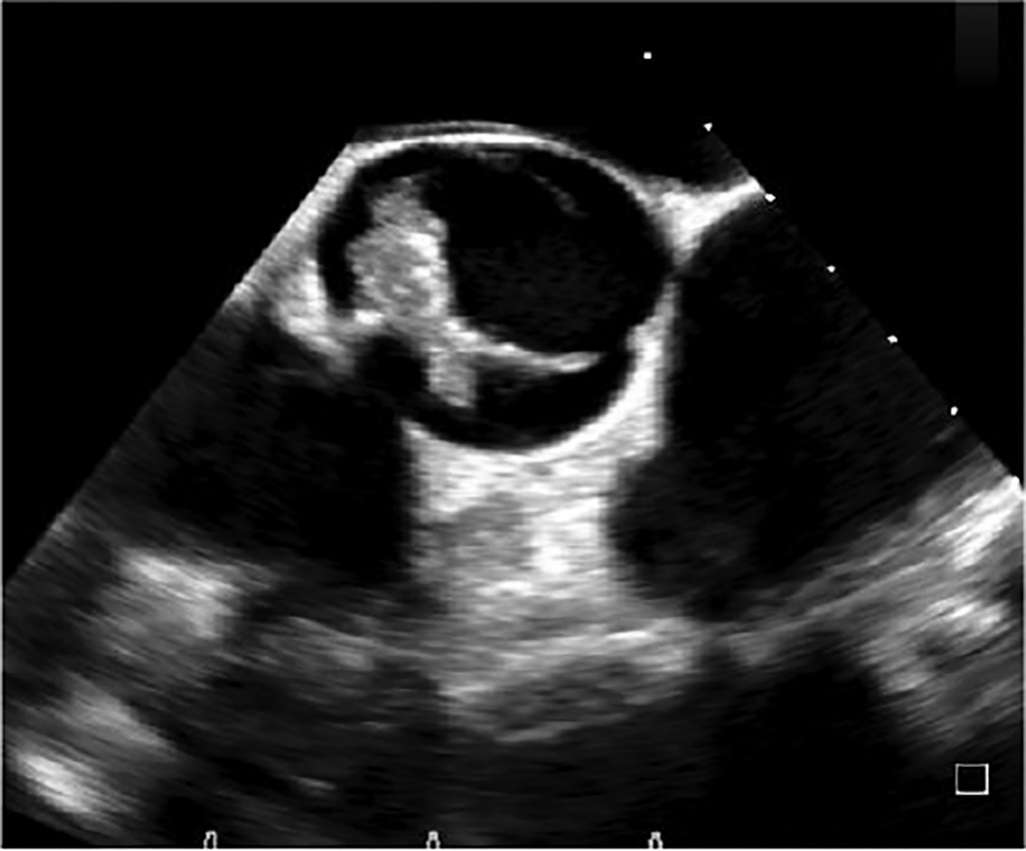
Bicuspid aortic valve (BAV).

**FIGURE 2:**
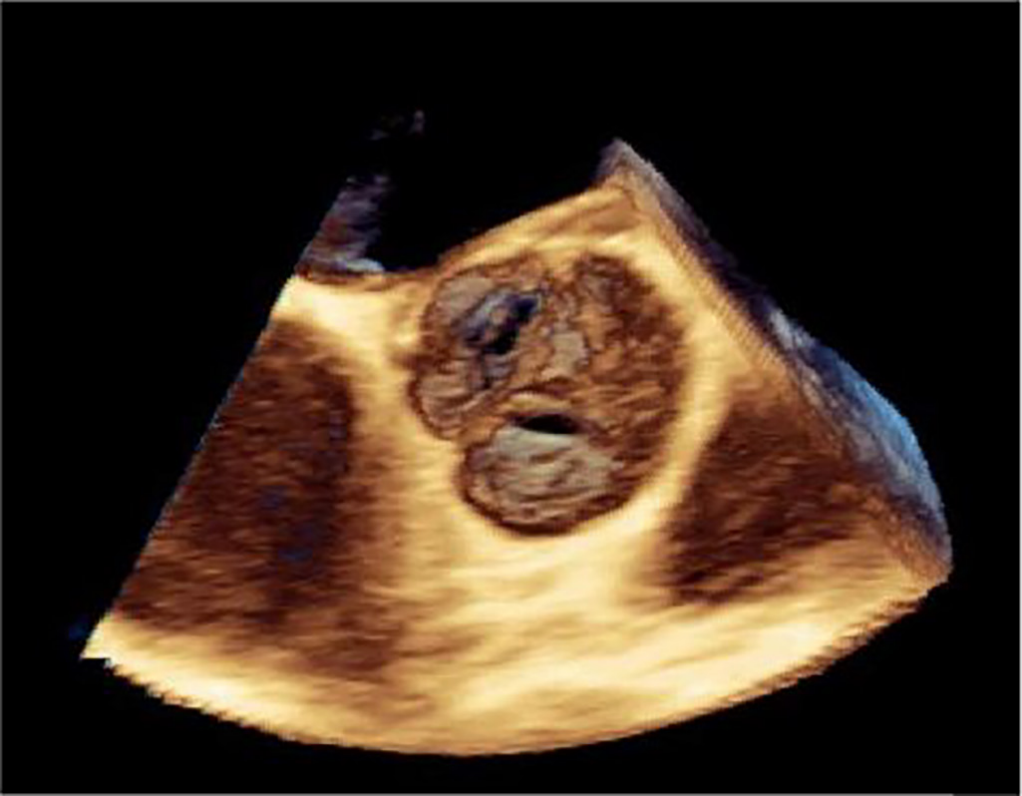
Massive vegetation in aortic valve.

**FIGURE 3:**
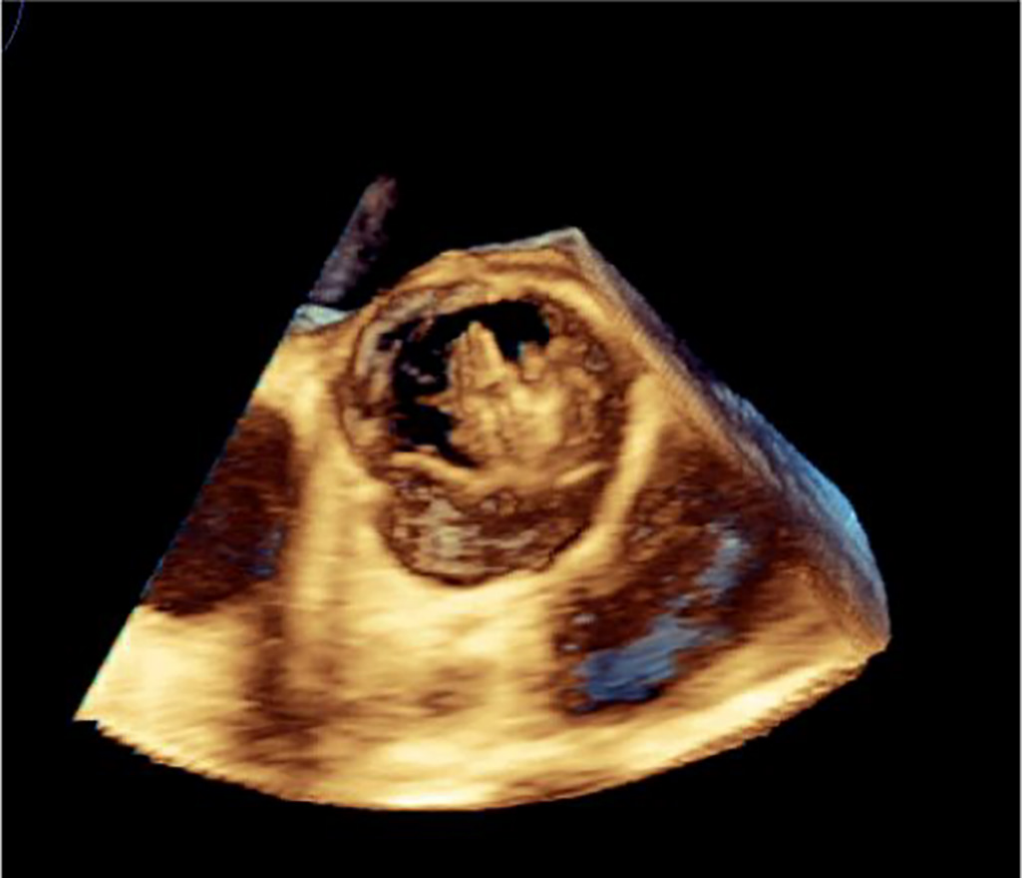
A mass (21 × 15mm) was seen in the ventricular side of right coronary cusp (RCC).

At the first visit to another hospital, Early blood tests showed: C‐reactive protein (CRP): 55 mg/dL, Erythrocyte sedimentation rate (ESR): 46 mm/hr, Leukocyte count (WBC): 7.48/μl, Serum Iron (Fe)a: 11 μg/dL, Hemoglobin (HB): 8.8 g/dL, Hematocrit (HCT): 27.4%, Mean corpuscular volume (MCV): 54.8 fL, Platelet(Plt): 145/μl. For further diagnostic evaluations, Wright and 2ME were requested, which were positive. In the following, he was admitted with the diagnosis of anemia and brucellosis due to positive Wright and Coombs tests. A complete abdominopelvic ultrasound showed just splenomegaly and hepatomegaly. For the evaluation of heart involvement, trans thoracic echocardiography was done for the patient, which reported ejection fraction (EF) =45%, endocarditis, and the presence of massive vegetation in the aortic valve. Due to the echocardiography report, he was sent to our center for Transesophageal echocardiography (TEE) and further treatment. Following the diagnosis, antibiotic therapy started as soon as possible and 1 week before the planned operation. Early tests in our center showed: Coombs‐Wright: 1/1280, Wright: 1/640, 2ME 1/160. It should be noted that a blood culture sample was also sent, which was negative. For further evaluation of the cardiac complication, TEE was performed which showed: bicuspid aortic valve (BAV) with severe Aortic insufficiency (AI) (Figure 1) & large highly mobile mass (21 × 15mm) was seen in the ventricular side of right coronary cusp (RCC) protrude to the aorta in systole (Figure 2 and 3).

### 2.2 Therapeutic intervention

**FIGURE 4:**
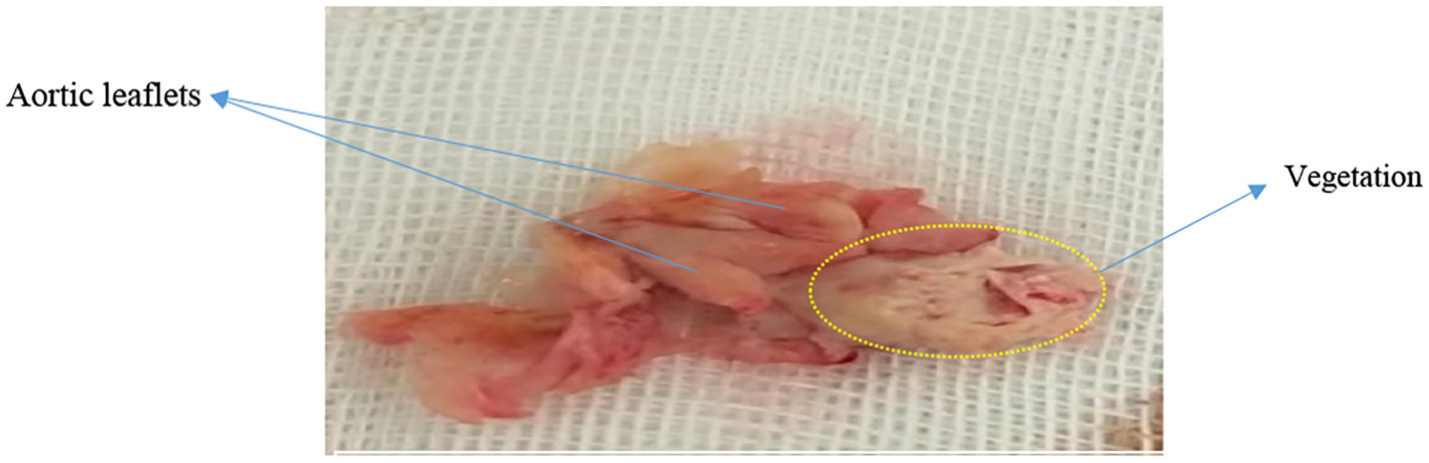
Massive vegetation on the surface of the valve.

Finally, the patient underwent surgery. Under general anesthesia and endotracheal intubation, the patient underwent a median sternotomy. After that, the aorta and right atrium were cannulated and cardiopulmonary bypass was established. Next, the aortic clamp was applied and cardioplegia was injected into the aorta. Then transverse aortotomy was performed and the rest of cardioplegia was injected into the both coronary ostiums; As a result, cardiac arrest was induced. Then, the aortic valve was exposed and due to the high destruction of the valve leaflets and massive vegetation, it was decided to replace the valve. Therefore, massive vegetation on the surface of the valve was removed (Figure 4) and the annulus was completely cleaned. Then, the aortic metallic valve was inserted by separate method. Then the aorta unclamped and the heart began to beat gradually and spontaneously (Aortic clamp time was 50 min and cardiopulmonary bypass time was 2 h). the patient was delivered to the cardiac surgery ICU with stable hemodynamics. After consulting with an infectious disease specialist, continuation of the antibiotic treatment that was started before the operation recommended and treatment continued for 12 weeks, streptomycin 1 gm intramuscular once daily for 3 weeks and doxycycline 100 mg twice a day plus tablet rifampin 900 mg once daily for 12 weeks. Surveillance of creatinine and liver enzymes level of serum was done twice weekly during first 3 weeks of drug treatment and weekly for remain duration of antibiotics use. The patient well tolerated the antibiotic regimen without need for adjustment of dosage.

### 2.3 Follow‐up and outcomes

In echocardiography after the operation, due to previous myocarditis, EF was 35%. For this reason, heart failure counseling was given. After heart failure treatment including tablet bisoprolol 5 mg daily and close monitoring, the patient's ejection fraction reached 60%. Due to metallic valve, he was treated with heparin after the operation. Then, oral warfarin was substituted and his INR was maintained between 2.5 and 3. After 2 weeks, He was discharged in good general condition. After 6 months, all of blood tests were normal and Echocardiography showed EF = 60% and no sign of any lesions.

## 3 DISCUSSION

With over 500,000 new cases reported each year and prevalence rates in certain nations reaching 10 cases per 100,000 people, brucellosis continues to be the most prevalent bacterial zoonosis in the world. Brucellosis continues to be underdiagnosed and underreported while being endemic in many developing countries.^11^ In many developing nations, including Iran, brucellosis continues to be one of the most difficult problems for health, and the economy to solve.^3^, ^11^ Nearly every province in Iran has a high prevalence of brucellosis; between 2009 and 2015, the trend grew from 34.6 to 71.4 cases per 100,000 people. The incidence and mortality rate have also increased, from 120.74 to 251.43 cases per 100,000 people, and from 198 to 412 cases per 100,000 people, respectively.^12^

Endocarditis caused by Brucella was discovered in 80% of fatal brucellosis patients, making it the most common cause of mortality from human brucellosis.^13^ Its rapid and extensive tissue destruction is related to its mortality; Delayed diagnosis or misdiagnosis of other diseases is also associated with high mortality.^14^

Patients with brucellosis typically experience non‐specific symptoms such fever, night sweats, arthralgia, headache, fatigue, anorexia, myalgia, and weight loss. Additionally, cardiac and neurological manifestations might make the diagnosis more challenging.^15^ Clinical manifestations of our case were insidious fever, generalized pain, weakness and lethargy, anorexia, and headache.

The diagnosis of brucellosis depends on the clinical presentations and laboratory tests. Detection of Brucella species by culture method is sometimes unsuccessful; therefore, serological tests are preferred; These tests are easy to perform, and results can be obtained within a short span of time.^16^, ^17^ For the diagnosis of human brucellosis, a number of serologic tests have been created, including the enzyme‐linked immunosorbent assay (ELISA), antihuman globulin (Coombs), indirect fluorescent antibody (IFA), and standard agglutination tube (SAT) test.^18^, ^19^ In our case, to confirm the diagnosis, we used Wright and Coombs tests.

Unlike other bacteria‐related endocarditis, Brucella endocarditis is characterized by fibrosis, hyalinization, and calcification. Heart failure is the main cause of death in Brucella endocarditis.^20^ Aortic valve involvement was described in 82% of cases with brucella endocarditis, but mitral valve involvement is less common.^10^, ^21^ In our case, he had heart failure and endocarditis with a massive vegetation in his aortic valve.

In certain clinical situations, a combined medical and surgical approach is necessary for the successful treatment. Development of cardiac failure, hemodynamic failure, big vegetation, the existence of emboli, valvula damage, and abscess formation is the main surgical indications in brucella endocarditis.^22^ In our case, Due to the high cardiac involvement, long‐term symptoms, massive vegetation in aortic valve and valve destruction, medical treatment alone is not considered.

The relapse rate of brucellosis was 6.6% until 9 months after starting the treatment; Therefore, treatment failure and relapse are major problems in the management of brucellosis. In developing countries, treatment with the oral combination doxycycline/rifampicin is preferred because of its convenience.^23^ Our patient was treated with doxycycline and rifampin after surgery to prevent relapse.

## 4 CONCLUSION

In dealing with patients who have clinical symptoms, recurrent fevers and chronic course, especially in areas where unpasteurized dairy products are used, brucellosis should be considered and further investigation should be done for the patient through paraclinical investigations. Rare complications like endocarditis are possible in patients diagnosed with Brucella infection, as brucellosis is endemic in Iran. We suggest that medical and surgical therapy together can be considered the best outcome in patients with severe Brucella endocarditis.

## AUTHOR CONTRIBUTIONS

**Seyed Mohsen Mirhosseini:** Conceptualization; project administration; supervision; writing – original draft. **Abdolhamid Bagheri:** Supervision; visualization. **Mehran Lak:** Supervision; visualization. **Zahra Ansari Aval:** Data curation; resources; supervision. **Mahdi Rezaei:** Methodology; writing – original draft.

## FUNDING INFORMATION

This research did not receive any specific grant from funding agencies in the public, commercial, or not‐for‐profit sectors.

## CONFLICT OF INTEREST STATEMENT

None.

## CONSENT

Written informed consent was obtained from the patient who participated in this study. This case report did not receive any funding. Authors have access to all source data for this case report.

## Data Availability

The data that support the findings of this study are available on request from the corresponding author. The data are not publicly available due to privacy or ethical restrictions.
